# Determinants of Delayed Treatment-seeking for Diarrheal Diseases among Mothers with under-five Children in North Western Ethiopia, 2020: A case-control Study

**DOI:** 10.4314/ejhs.v31i6.11

**Published:** 2021-11

**Authors:** Abebaw Getu Kbede, Mulunesh Alemayew, Yilkal Tafere, Getaneh Baye Mulu

**Affiliations:** 1 Department of Public Health, College of Health Sciences, Debre Markos University, Debre Markos, Ethiopia; 2 Department of Nursing, College of Health Sciences, Debre Berhan University, Debre Berhan, Ethiopia

**Keywords:** Diarrhea, Delayed Treatment-Seeking, Children, Ethiopia

## Abstract

**Background:**

Delays in seeking timely proper care pay a large number of deaths from diarrhea in children. Timely and appropriate health care seeking in under-five children with diarrhea reduces life-threatening complications. This study aimed to investigate determinants of delayed treatment-seeking for diarrheal diseases among mothers with under-five children.

**Method:**

In Debre Markos public health facilities, a facility-based case-control study was conducted among 412 mothers ((137 cases and 274 controls) from September 1 to October 15, 2020. Consecutive sampling was employed to select cases and controls. Data was collected using a semi-structured interviewer-administered questionnaire. Data were entered into Epi- Data version 4.2.1 and exported to STATA version 14 for analysis. Predictors with P-value <0.25 in the bivariable logistic regression model were candidates for multivariable logistic regression. Pvalue <0.05 was used to declare statistical significance. Finally, results were presented in the form of texts and tables.

**Result:**

From 412 selected participants, 408 mothers (136 cases and 272 controls) were included. Female children [AOR 1.85(95% CI 1.15–2.98)], Child age < 24 months [AOR 1.64 (95% CI 1.01–2.65)], mothers'/caregivers without formal education [AOR 4.61 (95% CI 2.03–10.44)], poorest wealth index category [AOR 4.24 (95% CI 1.90–9.48)], absence of health insurance [AOR 3.04 (95% CI 1.60–5.78)], and self-medication [AOR 3.6 (95% CI 1.75–7.4)] were determinants of delayed treatment-seeking.

**Conclusion:**

Being female, young age, educational status of the mother, lowest wealth index category, self-medication, and absence of health insurance were determinants of delayed treatment-seeking for diarrheal diseases. Preventive care programs should target age, low socioeconomic status, and a low educational class of the mother

## Introduction

Diarrhea is the passage of three or more loose or liquid stools per day (or more frequent passage than usual for the individual) (1). It remains a significant public health concern, contributing to 8% of deaths of under-five children worldwide (2). Among all from 5.4 million global deaths of under-five children, about 78% of under-five children's deaths were jointly shared by African and South Asia countries (3). In addition, Sub-Sahara Africa remains the country with the highest mortality rate of under-five globally, with 76 deaths per 1,000 live births (4). In many African countries, the median public health facility to treat common childhood illnesses in under-five children is below 50% (5).

Diarrhea is the world's leading cause of death among children under five, accounting for a considerable number of deaths globally. The majority of diarrhea-related deaths occurred in Sub-Saharan Africa and South Asia (6). Globally, there are nearly 1.7 billion cases of under-five children diarrhea disease, killing more than 525 000 children every year (7). Diarrheal death unfairly affects South Asia and Sub-Sahara Africa, which shared 91% in 2010 worldwide diarrhea deaths (6).

Healthcare policies and program planning require knowledge about healthcare-seeking behavior for early diagnosis, effective treatment. Early use of health services and enforcement with successful treatment will reduce morbidity, disability, and mortality (8).

In Ethiopia, seeking medical care in health facilities to treat common childhood illnesses varies from region to region. It ranges from 5 to 72%—nearly 73% of those seeking advice in government health care facilities(9, 10). Due to diarrheal illnesses and long-term complications, childhood mortality was reduced by 20 percent if mothers early sought appropriate and prompt care(3). However, the habit of healthcare-seeking behavior was relatively poor; thus, only a tiny proportion of under-five children receive appropriate treatment timely(10–17). According to the 2016 Ethiopia Demographic and Health Survey (EDHS) report, only 44% of under-five children with diarrheal were received advice or treatment in the health facility. Thus, despite the need for health care, it has often been delayed ever reaching a health facility, contributing to the morbidity and mortality of a significant number of under-five children. In addition, it would lead to complications in the long term(10, 13, 15–17).

In 2003 the Ethiopian government initiated the Health Extension Program (HEP). At least two Health Extension Workers were deployed at each kebele, delivering prevention, health promotion, and curative service at the health post through the Integrated Community Case Management (ICCM) program. They also worked to improve the health-seeking behavior of mothers/caregivers of children under five with common childhood diseases (18). As a result, care-seeking behavior has also improved, rising from 32% in 2011 to 44% in 2016 (18, 19).

While Ethiopia has made significant progress in reducing under-five child mortality, 67 under-five children die per 1,000 live births, and 480 children die every day from easily preventable diseases in 2018(20). In addition, immediate treatment within 24 hours was low where acute diarrhea was recognized among mothers/caregivers of children seeking care. Ethiopia's scientific studies showed that 27% of treatment was obtained promptly within 24 hours after acute diarrhea was recognized, and 12.2% treatment was obtained after 24 hours of recognizing critical diarrhea illness (11).

Inability to identify situations that endanger life and poor care-seeking behaviors caused that delay treatment-seeking for the caregivers. This delay may affect children's health and result in complications that make medical treatment less safe and ineffective(13, 21).

Treatment for common childhood disease illnesses like diarrhea is very successful when treatment is sought. Thus, morbidity and mortality from this disease can be reduced when early care is sought. Caregiver's ability to recognize and seek prompt care for these common childhood diseases is instrumental in reducing child deaths in low-and middle-income countries (LMICS). The importance of the ability of parents to identify and find prompt treatment for their children is also one of the key recommended activities in the Global Pneumonia and Diarrhea Control Action Plan of the WHO and UNICEF (22).

Having up-to-date evidence for the factor affecting the timely healthcare-seeking behavior of mothers of under-five children with diarrheal diseases is essential for planning intervention strategies, improving treatment compliance, evaluating health care services in the study area. Therefore, this study aimed to identify determinant factors of delay in timely care-seeking for diarrheal diseases among mothers with under-five children who visit the public health facility of Debre Markos Town.

## Methods

**Study design, area and setting**: A facility-based unmatched case-control study design was conducted in the public health facilities of Debre Markos Town from September 1 to October 15, 2020.

All mothers of under-five children with diarrheal illness utilized health care services from Debre Markos town public health facilities used as source population.

**Sample size determination**: The sample size was determined using Open Epi version 7.2.1 using double population proportion exposure difference formula by considering significant determinant variables (child age in months, preferred health facility, selecting the mothers' health facility, and educational status). Considering child age (23) in a month was taken as the primary exposure variable since it yields a larger sample size, with AOR 1.9, 387 with the proportion of exposure among controls 44%, 80% of power, 95% confidence level, 1 to 2 case to control ratio the estimated sample size was 375. By adding 10% of the non-response rate, the final sample size was 412(137 cases and 274 controls).

**Cases**: All mothers of under-five children with diarrheal illness sought treatment after 24 hours of recognizing signs/symptoms of diarrhea during the data collection period.

**Controls:** During the data collection period, all mothers of under-five children with diarrheal illness sought treatment within 24 hours of recognizing signs/symptoms of diarrhea.

**Eligibility criteria**: Those mothers of under-five children seeking diarrheal illness and who visited pediatric and IMNCI clinics during the study period were included. In contrast, those mothers of under-five children with diarrheal disease who visited the health facility for follow-up of diarrhea disease treatment were excluded from the study.

**Sampling procedure**: All five public health facilities: (Debre Markos Referral Hospital, Debre Markos HC, Gozamen HC, Wiseta HC, and Hidase HC) found in Debre Markos town were included. The data were collected consecutively from all five public health facilities until the total sample size was obtained. The cases were the participant who came to the health facility after 24 hours of the onset of diarrheal illness whereas the controls were under-five children with diarrheal disease who came to the health facility within 24 hours of the start diarrheal disease. When mother's compliance with diarrhea in their child has completed their consultation with a health care professional, they moved to a private room for an interview until the total required sample size was obtained.

While treatment-seeking (Delay/Timely) was dependent variable, **independent variable were- Predisposing factor** (age of child, age of mothers, sex of a child, educational status of the mother and the father, marital status of the mothers, residence, occupation of mothers, and father); **enabling factors** (wealth index, treatment costs, health insurance, distance to the nearest health facility, preferred health facility, nearby health facility, and reason for the selected facility); **need/disease factor** (symptoms with current diarrhea, dehydration status, last 6-month visit, self-medication, traditional medicine, and diarrhea types); **health system-related factors** are (client perception concerning the health care professional).

**The following operational and term definitions were used.**

**Wealth index:** is a composite measure of a household's cumulative living standard. First, the household wealth status of the study participant was assessed through principal component analysis. Then the participant was categorized into five categories, the poorest, the poor, the middle, the rich, and the richest. Finally, the factor that explains the most significant variance in the variables was included(24).

**Caregiver**: any person above 18 years of age who is directly responsible for the child's care at the study time (25).

**Treatment seeking.** Any treatment was sought from a defined governmental health facility for a child with diarrheal disease.

**Treatment delay**. Care or treatment sought from health facilities after 24 hours from recognizing the presence of diarrhea in underfive children(23, 26).

**Treatment seeking timely**. Health facilities seek care or treatment within 24 hours from recognizing diarrhea in under-five children(23, 26).

**Self-medication**. Purchasing and utilizing medicine from a pharmacy or shops without a prescription.

**Traditional medicine**. Experience-based knowledge, skill, and practice are applied to treat apparent illness and sickness patients by traditional healers, herbalists, and magicians.

**Client perception towards respect of health workers**: The respect of health workers was measured in terms of the patient's response to the five questions provided. The questions were organized on a Likert scale, ranging from 1(strongly disagree) to 5 (strongly agree). Then, the mean score was calculated, and those who score mean and above the mean were considered as they get respect from health workers, and those who score below the standard were thought that as they did not get care from health workers (27).

**Data collection tools and procedure:** Data was collected using a semi-structured interviewer-administered questionnaire adapted from EDHS-2016 (24). It included predisposing, enabling, need/disease factors. One BSc Nurse collected the data in Debre Markos referral hospital. In addition, four diploma Nurses in health centers who were not assigned in under-five children's clinics to provide the service for children presenting with the medical case were collected. Two BSc Nurse supervised data collectors daily to maintain the quality of data.

**Data quality control:** The questionnaire was adapted from EDHS-2016 and prepared initially in English. It was translated into Amharic and back to English to check language consistency. The training was given to data collectors and supervisors about selecting study participants, interviewing methods, and checking the already filled questionnaire by the principal investigator. Pretest was conducted at Emanuel health center other than the study area on 5% of a sample size to check consistency and any ambiguity of the questionnaire.

**Data processing and analysis:** Before the analysis, data were cleaned and coded. Data was entered using Epi-Data version 4.2.1 and analyzed using STATA version 14 statistical software. Socio-demographic profiles of variables frequency distribution, summary measures such as median, range, and standard deviation were calculated. Factors that showed a statistically significant association (p-value less than 0.25) with the dependent variable in the bivariable models were considered candidate variables for the multivariable logistic regression models. To minimize adjustment bias, factors of delay in seeking health facilities were fitted into two multivariable models. In the final multivariable models, the level of multicollinearity was checked and fitted using variance inflation factor (VIF). In the final multivariable models, the goodness of fit, Hosmer Lemeshow was checked and fitted for the data with the P-value of 0.094. P-value less than 0.05 was considered statistically significant in multivariable logistic regression. Finally, results were presented in the form of text and tables.

## Results

**Scio-demographic characteristic of study participant**: In this study, from 412 sampled populations, 408 mothers of under five children with diarrheal disease (136 cases and 272 controls) were included, which made the response rate 99%. More than half, 84 (61.7%) of the cases were children < 24 months, and nearly half, 133(48.9%) of control were children < 24months. The child's age ranges from 2 to 59 months, with a mean age of 21(±13.3 SD) months. More than half of respondents were females, 83(61%) among cases, and males, 146(53.6%) among controls. Nearly two-thirds of 92(67.6%) cases and 155(57%) control mothers/caregivers were between the age group of 26–34 years (Table 1).

**Table 1:** Scio-demographic factors of delayed treatment-seeking among mothers of under-five children with diarrheal disease visited Debre Markos Town public health facilities, Northwestern Ethiopia, 2020(n=408)

Variables		Cases(N=136)		Controls(N=272)	
	Categories	N	%	N	%
Ages of child	<24months	84	61.7	133	48.9
	≥24months	52	38.3	139	51.1
Sex of child	Females	83	61	126	46.3
	Males	53	39	146	53.7
Place of residence	Rural	18	13.3	34	12.5
	Urban	118	86.7	238	87.5
birth order of child	≤2	88	64.7	186	68.4
	3–4	41	30.1	70	25.7
	≥5	7	5.2	16	5.9
Age category of the mother	15–25years	30	22	70	25.7
	26–34years	92	67.6	155	57
	≥35years	14	10.4	47	17.3
Educational status of mothers	No formal education	35	25.8	53	19.5
	Primary school	61	44.8	76	29.4
	Secondary school	20	14.7	59	21.7
	College and above	20	14.7	84	30.9
Educational status of the father	No formal education	11	8	17	6.2
	Primary school	46	33.8	64	23.5
	Secondary school	17	12.6	37	13.6
	College and above	62	45.6	154	56.7
Occupation of mothers	Housewife	85	62.5	152	55.9
	Government employee	16	11.7	51	18.75
	Merchant	20	14.7	48	17.6
	Farmer	15	11	21	7.7
Occupation of father	Government employee	46	33.8	119	43.75
	Merchant	51	37.5	85	31.25
	Farmer	18	13.3	30	11
	Daily laborer	21	15.4	38	14
Ethnicity of mothers	Amhara	134	98.5	269	99
	Tigray	2	1.5	3	1
Marital status of the mother	Single	3	2.2	19	7
	Married	124	91.2	243	89
	Divorced	9	6.5	10	4
Religion of mothers	Orthodox	125	92	245	90
	Muslim	5	3.7	14	5
	Protestant	5	3.7	10	4
	Catholic	1	0.7	3	1

Note: N- number

**Enabling factor**: Forty-six (33.8%) cases belong to the poorest wealth index, while 64 (23.5%) controls were among poor wealth index groups. Nearly two-third, 101(74.2%) cases and 232(85.2%) controls respond that the cost of treatment at a health facility was easy. Eight-five (62.5%) cases and 146 (53.6%) controls homes were at a distance of 15–30 minutes from the preferred health facility on foot (Table 2).

**Table 2:** Enabling factors of delayed treatment-seeking among mothers of < 5 years children with diarrheal disease visited Debre Markos Town public health facilities, Northwestern Ethiopia, 2020 (n=408)

Variables	Responses	Cases(N=136)		Controls(N=272)	
		N	%	N	%
Wealth index	Poorest	46	33.8	62	22.8
	Poor	40	29.4	64	23.5
	Middle	18	13.2	46	16.9
	Rich	18	13.2	50	18.4
	Richest	14	10.3	50	18.4
Cost of treatment	Easy	101	74.3	232	85.3
	Difficult	35	25.7	40	14.7
Member of health insurance	Yes	20	14.7	70	25.7
	No	116	85.3	202	74.3
Distance of health facility by foot	<15	20	14.7	44	16.2
	15–30 minutes	85	62.5	146	53.7
	30–60 minutes	17	12.5	64	23.5
	60–120 minutes	12	8.8	13	4.8
	> 120 minutes	2	1.5	5	1.8
Preferred HF	HC	133	97.8	35	12.9
	Hospital	3	2.2	237	87.1
Nearby HF	HC	109	80.1	206	75.7
	Hospital	27	19.9	66	24.3
Reason for preferred HF	Do not charge too much	35	25.7	59	21.6
	Nearness	93	68.3	157	57.7
	Respect given	47	34.5	124	45.5
	Examination given	22	16.17	71	26.10
	Low waiting time	79	58.08	139	51.10
	Treatment is effective	2	1.4	7	2.5
	Always open	4	2.9	20	7.3
	Medications available	3	2.2	26	9.5
Who decides first to take the child to the HF	Mother	100	73.5	157	57.7
	Father	35	25.8	105	38.6
	Grandfather	1	.7	10	3.7

NB: HF: Health facility; HC: Health center

**Need/Disease and health system-related characteristics**: According to this finding, the dehydration status of under-five children with diarrhea illness 17(12.5%) cases and 26(9.6%) controls had severe dehydration. Approximately 25(18.3%) of the case and 29(10.6%) controlled developed diarrhea and visited a health facility within the last six months. Regarding the type of diarrhea, 65(47.7%) of mothers/caregivers of the case group and 137(50.3%) of mothers/caregivers of the control group complained of watery type of diarrhea (Table 3).

**Table 3:** Need/disease and health system-related factors of determinants of delayed treatment-seeking among mothers of under-five children with diarrheal disease visited Debre Markos Town public health facilities, Northwestern Ethiopia, 2020 (n=408)

Variables	Categories	Cases(N=136)		Controls(N=272)	
		N	%	N	%
Dehydration status	No dehydration	45	33.1	178	65.4
	Some dehydration	74	54.4	68	25
	Severe dehydration	17	12.5	26	9.6
Self-medication	Yes	27	19.9	19	7
	No	109	80.1	253	93
Tradition medicine	Yes	1	.7	5	1.8
	No	135	99.3	267	98.2
Diarrhea in last 6-month visit	Yes	25	18.4	29	10.7
	No	111	81.6	243	89.3
Types of diarrheas	Blood	21	15.4	71	26.2
	Mucoid	50	36.7	64	23.5
	Watery	65	47.7	137	50.3
Symptoms associated with current diarrhea	Vomit	22	16.1	79	29.0
	Feed poorly	20	14.7	55	20.22
	Fever	17	12.5	26	9.5
	Sunken eyeball	4	2.9	3	1.1
	Restlessness	3	2.2	9	3.3
Client perception on respects of HCP	Good	67	49.2	110	40.4
	Poor	69	50.8	162	59.6

HCP: Health care professionals

**Determinants of delay in treatment seeking**: As to this study, age of the child, sex of the child, educational status of mother/caregivers, educational status of the father, wealth index category, health insurance, self-medication and client's perception on respects of health care worker) were computed in multivariable logistic regression. Of these, sex and age of child, mother/caregiver educational status, wealth index, health insurance, and self-medication were statistically significant for delayed treatment-seeking among mothers/caregivers of under-five children with diarrheal disease.

Mothers/caregivers of female under-five children were [AOR 1.85 (95% CI 1.15–2.98), P =0.011] times more likely to delay treatment-seeking for their diarrheal disease than mothers/caregivers of male children. Mothers/caregivers of young children with <24months were 1.6 times more likely to delay than mothers of older children with >24months [AOR1.64 (95% CI 1.01–2.65), P =0.042]. Mothers/caregivers of under-five children with diarrheal disease who did not attend formal and primary education were five times more likely to delay in treatment-seeking with the diarrheal disease to that of mothers/caregivers who attended college and above education respectively [AOR 4.6 (95% CI 2.03–10.44), P =0.01] and [AOR 4.56 (95% CI 2.25–9.27), P =0.01].

Regarding household wealth status, mothers with under-five children with diarrheal disease belonged to the poorest, and poor were four times more likely to delay in treatment-seeking for their children with the diarrheal disease compared to most affluent wealth index category [AOR 4.24 (95% CI 1.90–9.48), P = 0.01] and [AOR 3.8 (95% CI 1.71–8.67), P = 0.001] respectively. Mothers with under-five children with the diarrheal disease who was not a member of health insurance were six times more likely to delay than their counter parts with [AOR 3.04 (95% CI 1.60–5.78), P =0.001)].

Mothers who used self-medication for an under-five child with the diarrheal disease were four times more likely to delay as compared to those who were not using self-medication [AOR 3.6 (95% CI 1.75–7.40), P =0.01] (Table 4).

**Table 4:** Bi-variable and Multivariable Logistic Regression analysis of determinants of delayed treatment-seeking among mothers of under-five children with diarrheal disease visited Debre Markos Town public health facilities, Northwestern Ethiopia, 2020 (n=408)

Variables	Categories	Cases	Controls	COR (95%CI)	AOR (95%CI)
		N (%)	N (%)		
Child age	<24months	84 (61.7)	133 (48.9)	1.7(1.1–2.6)	1.64 (1.01–2.65) *
	≥24months	52 (38.3)	139 (51.1)	1	1
Sex of child	Females	83 (61)	126 (46.3)	1.8 (1.2–2.75)	1.85 (1.15–2.98) *
	Males	53(39)	146 (53.7)	1	1
Educational status of father	No formal education	11 (8)	17 (6.2)	1.6 (0.7–3.6)	1.7(0.6–4.55)
	Primary school	46 (33.8)	64 (23.5)	1.8 (1.1–2.9)	1.6 (0.87–2.94)
	Secondary school	17 (12.6)	37 (13.6)	1.14 (0.6–2.2)	0.91 (0.4–2)
	College and above	62 (45.6)	154 (56.7)	1	1
Educational status of mothers	No formal education	35 (25.7)	53 (19.5)	2.8 (1.45–5.3)	4.6(2.03–10.4) **
	Primary school	61 (44.8)	76 (27.9)	3.4 (1.9–6)	4.56(2.2–9.3) **
	Secondary school	20 (14.7)	59 (21.7)	1.4 (0.7–2.9)	1.63(0.75–3.5)
	≥ College	20 (14.7)	84 (30.9)	1	1
Health insurance	Yes	20 (14.7)	70 (25.7)	1	1
	No	116 (85.3)	202 (74.3)	2 (1.2–3.5)	3 (1.6–5.8) **
Self-medication	Yes	27 (19.9)	19 (7)	3.3 (1.75–6.2)	3.6 (1.75–7.4) **
	No	109 (80.1)	253 (93)	1	1
Wealth index	Poorest	46 (33.8)	62 (22.8)	2.6 (1.3–5.4)	4.2 (1.9–9.5) **
	Poor	40 (29.4)	64 (23.5)	2.2 (1.09–4.6)	3.85 (1.7–8.7) **
	Middle	18 (13.2)	46 (16.9)	1.4 (0.6–3.1)	1.8 (0.7–4.3)
	Rich	18 (13.2)	50 (18.4)	1.3 (0.6–2.86)	1.6 (0.66–3.9)
	Richest	14 (10.3)	50 (18.4)	1	1
Client perception on respect of HCP	Poor	67 (49.2)	110 (40.4)	0.7 (0.5–1.05)	0.8 (0.5–1.3)
	Good	69 (50.8)	162 (59.6)	1	1

* Statistically significant with a P-value ≤ of 0.05

HCP: Health care professionals

## Discussion

The objective of this study was to identify determinants of delayed treatment-seeking among mothers with children under five with diarrheal disease.

This study revealed that female children's mothers were two times more likely to delay treatment-seeking than male children's mothers/caregivers [AOR 1.85 (95% CI 1.15–2.98)]. The result aligns with the study conducted in Arbaminch, Woliso, Ethiopia, and Uganda (23, 26, 28). Thus, gender inequality that systematically affects females in the community could be the possible reason for the difference in seeking care between male and female children; this can lead mothers to pay attention only to the male. (29, 30). Conversely, this study differs from studies conducted in Niger and Sierra Leone in which gender was not associated with delay in treatment-seeking(31, 32).

This study also revealed that treatment-seeking for the diarrheal disease is predicted by age. Young children's mothers/caregivers (<24months) were one and half times more likely to delay treatment-seeking for diarrheal disease than mothers of older children (≥24months) [AOR 1.64 (95% CI 1.01–2.65)]. This result is consistent with central and southern Ethiopia and Niger (23, 26, 31). This is because mothers link diarrhea to teeth eruption, resulting in mild and self-limited diarrhea in younger children. Nevertheless, this finding contradicts the Global report on seeking treatment and access to health services (30, 33).

Furthermore, mothers who did not attend formal education and attending primary school were four-point six times and four-point five times more likely to delay than those who attended college and above with [AOR 4.6 (95% CI 2.03–10.44)] and [AOR 4.56 (95% CI 2.25–9.27)] respectively. This trend has been shown similarly in previous studies conducted in Woliso, Arbaminch, and Malaysia (23, 26, 30). It is thought that mothers/ caregivers who do not have formal schooling or attending primary school are less likely to notice severe symptoms and less likely to have financial resources to get the sick child timely to the health facility. So, they are more likely to wait for diarrhea improvement alone at home. Moreover, without formal education, the mother may not have a basic understanding of the effects of delayed treatment-seeking for children with diarrheal disease(16, 34, 35). This result is contrary to a study conducted in Niger (31). This difference might be due to different study areas and times.

Moreover, these findings indicate that the odds of delayed treatment-seeking among poorest and poor wealth index category mothers/caregivers were four times more likely to delay than those who belong to the wealthiest index category [AOR 4.24 (95% CI 1.90–9.48)] and [AOR 3.8 (95% CI 1.71–8.67)] respectively. This study finding is consistent with the study conducted in Arbaminch (26), slums of Addis Ababa (11), and Malaysia(30). This might be due to a lack of assets to cover the expense of user fees/medicine, travel costs to and from health facilities (12, 33). However, this finding contradicts the study done in Woliso of central Ethiopia, Jeldu district of Ethiopia, and Kigali Rwanda(23, 34, 36). This may be due to a difference in respondents' ability to use these health services to pay user service fees or medication costs in these fields of study.

This study also revealed that for mothers who used self-medication at home for childhood diarrheal treatment, the odds of delayed treatment-seeking were four times higher than their counterparts [AOR 3.6 (95% CI 1.75–7.4)]. This finding was supported by a study done in Pakistan and Rwanda (36, 37). This might be because the mothers may get the drugs from shops which leads to wrong medication, and the child may not get the proper treatment timely.

Health insurance was also an important determinant of delay in treatment-seeking in this study.

Mothers of under-five children with diarrhea who were not health insurance members were three times more likely to delay treatment-seeking than their counterparts [AOR 3.04 (95% CI 1.60–5.78)]. However, the effects of this variable were not studied previously.

This study revealed that children aged (<24months), being female, low educational status of the mother, poorest wealth index category of the household, use self-medication, and not a member of health insurance were determinants of delayed treatment-seeking among mothers of under-five children with diarrheal disease. Preventive care programs should target the age, low socioeconomic, and a low educational class of the mother.
